# Mr. Annesley on Elongation and Unnatural Positions of the Colon

**Published:** 1830-04-01

**Authors:** 


### LVII.

### MADRAS MILITARY HOSPITAL.

### ON ELONGATION AND UNNATURAL POSITIONS OF THE COLON. By JAMES ANNESLEY, Esq.

In the second volume of Mr. Annesley's splendid work on Indian Diseases, there is a section under the above title, containing several interesting cases, some of which we shall here notice.

In ten of the plates there are representations of these unnatural positions

—some of them perhaps the original course of the colon, in other words, congenital—others evidently the result of functional disorder running into organic change. Mr. A. thinks it not improbable that accumulations formed in the lower part of the colon, and the restraint which is often put upon the inclination to stool, may induce irregular flexures and displacements of portions of that gut, and even an elongated state of it. He thinks also that the supervention of these consequences is favoured by a relaxed state of the mesocolon, the peritoneal covering of the bowels, and more especially of the longitudinal bands. We often find, in cases of old hernia, considerable displacement and elongation of the colon, and a stretched appearance of the peritoneum and mesentery; the parietes of the bowels being free from morbid change. The impaction, he thinks, of hard fæcal matters about the sigmoid flexure of the colon may produce a condition analogous to the above, and that this part of the gut may be carried lower into the iliac region or into the pelvis by the increased action of the parts above. When unnatural flexures are thus formed, or natural ones increased, morbid accumulations are more readily and more frequently produced—and, when once formed, very dangerous diseases of the colon itself, and of the neighbouring viscera often supervene. It is true that, during the life of the patient, we have no symptoms indicating the existence of this condition, which may not equally proceed from other kinds of disorder. The treatment, however, which is applicable to this derangement is also appropriate to others seated in the same viscus which are manifested by similar signs and characterised by alvine obstructions. We shall briefly notice one of the cases detailed by our author.

CASE 1. DISPLACEMENT OF THE CÆCUM AND COLON.

J. Clayton, aged 19 years, a soldier in the Madras European Regiment, July 1819, complained of troublesome cough and expectoration, acute pain in his chest on coughing, very small pulse, skin natural, giddiness, tongue moist and loaded. He had been ailing for six months, and had common remedies applied. He was attacked the evening before the date of report with vomiting and purging of green matter. Calomel was administered, leeches were applied to the sternum, and various means were used; but the symptoms above-mentioned continued, and indeed increased, till death put an end to his sufferings on the 7th of August. The dissection we shall give in Mr. Annesley's words.

"A considerable degree of vascular action appeared over the whole intestinal canal, particularly in the lower part of the ilium. The cæcum was thrown completely out of its place into the centre of the pelvis, immediately over the pubes, and was very much distended with flatus. The ilium was drawn round into the right iliac region, occupying the place of the cæcum, and entering it on the right side. On laying open the cæcum and caput coli, we found a membranous septum between the cæcum and head of the colon, which, although it did not occasion a complete obstruction, yet the passage must have been considerably interrupted. The coats of the intestine were covered with very dark spots, but there was no ulceration. From the sigmoid flexure to the extremity of the rectum there was considerable inflammation on the external coat; and on laying open and exposing the internal surface of this part of the bowel, it was found covered with similar spots to those which were observed at the head of the colon: there was no ulceration, but the coats were of a gristly hardness. The small intestines were generally of a healthy state; but in the peritoneal coat there was the appearance of considerable vascular action, though not amounting to inflammation. The external coat of the stomach was not in any way changed; but in the villous coat there was considerable vascularity, and about two or three inches from the cardia there was a spot of a verdigrise-green colour, which appeared more like a stain than sphacelation, the part being of firm consistence. The gall-ducts were open, and we were enabled to pass a probe through them into the gall-bladder, which was loaded with dark-coloured, thick bile. The liver was in general healthy, though there appeared some congestion in the right lobe. The kidneys, spleen, and pancreas, were natural. The mesenteric glands were enlarged. In the lungs there was much congestion in the posterior part, perhaps from gravitation of blood. The heart, externally, appeared natural; but on laying open the left auricle and ventricle, we found both loaded with a mass of coagulable lymph, which was so intimately interwoven in the columnæ carneæ, that it must have deprived the heart of the power of receiving its proper quantity of blood; and this may account for the singular oppression and want of pulse described during the treatment of the case. In the head there were signs of some congestion, and considerable arterial action, but no water."

A number of other cases follow, but we are unable to analyze them in this place.

---

